# Investigating the role of neuropathic pain relief in decreasing gait variability in diabetes mellitus patients with neuropathic pain: a randomized, double-blind crossover trial

**DOI:** 10.1186/1743-0003-11-125

**Published:** 2014-08-20

**Authors:** Surshen Karmakar, Houman Rashidian, Cynthia Chan, CaiXia Liu, Cory Toth

**Affiliations:** Department of Clinical Neurosciences, HMRB 155, Foothills Hospital, University of Calgary, Hotchkiss Brain Institute, 3330 Hospital Dr. NW, Calgary, AB T2N 4 N1 Canada

**Keywords:** Type 2 diabetes mellitus, Gait, Diabetic neuropathy, Diabetic neuropathic pain, Pregabalin

## Abstract

**Background:**

Subjects with diabetes mellitus (DM) develop gait dysfunction contributing to falls, reluctance to perform activities and injuries. Neuropathic pain (NeP) related to diabetic peripheral neuropathy (DPN) is associated with increased gait variability that may contribute to gait dysfunction. We used a portable device (GaitMeter™) and related gait and balance measures to measure gait parameters in painful DPN (PDPN) subjects prior to and during analgesia. Our hypothesis was that PDPN subjects would have decreased gait step variability when receiving pharmacological relief of NeP.

**Methods:**

DPN subjects with at least moderate NeP were assessed in a randomized, double-blind crossover study of pregabalin versus placebo. The outcome measure was variability in step length and step velocity. Testing for Timed Get-Up-and-Go Test, Tinetti Mobility Scales, Sway Testing, a Physiological Profile Approach, and fall-related surveys were also performed. DPN severity was quantified using the Utah Early Neuropathy Score.

**Results:**

PDPN subjects developed increased, rather than decreased, step length and step velocity variability during pregabalin treatment. There were no significant differences between cohorts for other physiological gait and balance testing. Non-significant NeP relief occurred in the pregabalin phase of study as compared with placebo. There was a negative relationship for step length with pain severity.

**Conclusion:**

Analgesia did not decrease gait variability in PDPN patients, and in fact, increased gait variability was seen during pregabalin treatment. Other important relationships of gait dysfunction with PDPN should be sought.

**Electronic supplementary material:**

The online version of this article (doi:10.1186/1743-0003-11-125) contains supplementary material, which is available to authorized users.

## Introduction

A strong predictor for falling is the presence of diabetes mellitus (DM) [1, 2]. Falling may occur in DM patients due to its many systemic complications including hypoglycaemia, orthostatic hypotension, obesity, cardiovascular disease, vestibular dysfunction, visual impairment, cognitive impairment [3], and age-related decline. However, another important contributor may be diabetic peripheral neuropathy (DPN), present in up to half of DM causing sensory, motor and autonomic dysfunction [4] impacting upon gait function [5].

Within DM subjects, those with DPN are more likely to suffer falls [6]. Although possibly related to reduction in proprioceptive input, other factors such as neuropathic pain (NeP) related to DPN may play roles. NeP, defined as pain initiated by a lesion or dysfunction of the nervous system [7], is present in 25-50% of DPN patients [8]. NeP in DPN (PDPN) leads to an “antalgic gait” [9], assumed in order to circumvent or decrease pain while walking. Antalgic gait is associated with reduced gait velocity, shorter cadence and step length, with less rhythmic accelerations [10]. Although initially appearing to be a more stable gait, an antalgic gait may actually have greater gait variability and higher risks of falling [11]. Gait variability, defined as the extent to which gait parameters such as step length or step velocity diverge from a mean value, has been previously investigated by our group with reference to PDPN. We have previously hypothesized that gait dysfunction, defined as abnormal or impaired functioning of walking or running, may be associated with greater extents of gait variability [9]. Our previous work has demonstrated that variability of step length and velocity increases in PDPN subjects when compared to a cohort of painless DPN subjects and control subjects [9], even though other forms of chronic pain, such as with low back pain, can contribute to reduced variability [12]. Thus, we postulated that NeP, a potentially treatable risk factor, is contributory to gait variability, gait dysfunction, and the subsequent risk of falling.

We hypothesized that patients with PDPN would have decreased gait variability with pharmacological NeP management using pregabalin, an indicated therapy for PDPN. If pain due to PDPN is itself responsible for the increased gait variability, then it could be theorized that reduced pain severity would reduce such variability to levels seen in DPN patients without pain. We performed a randomized, double-blinded, cross-over study comparing the effect of pregabalin, an effective pharmacotherapy for PDPN, compared with placebo intervention. Gait variability was assessed using GaitMeter™, a wireless, portable gait detection system previously studied in control subjects, DM patients, and patients with DPN [9], with reliability for its use in determining gait variability.

## Methods

### Subject recruitment

This study was a single-center, double-blind, randomized, placebo-controlled cross-over clinical trial. Two periods of six-week flexible-dose trials of pregabalin/placebo were compared using the same subject as comparator, with two week washout phases intervening. Ethical approval for this study was received from the University of Calgary Centre for Advancement of Health. Participant recruitment took place from August 2011-January 2013 using poster recruitment at the tertiary care clinics within the University of Calgary. Informed written consent was received from all participants prior to involvement. Each subject had pre-existing type 2 diabetes, based upon Canadian Diabetes Association guidelines. Further clinical and laboratory evaluation to verify presence of DPN occurred. Age of DM diagnosis, duration of DPN, and use of anti-diabetes treatments and other medications were documented. Any history of other systemic illness, toxic and medication exposure, alcohol use, and familial neuropathy was determined. All recruited subjects performed laboratory testing at study initiation and completion to identify other peripheral neuropathy causes [13]. Hemoglobin A1C, electrocardiography and hepatic and renal function blood work were performed at the beginning and end of each intervention period.

### Subject assessment

During baseline week, assessments and questionnaires were completed. Daily pain severity and associated clinical reporting diaries were initiated at baseline week, at the end of first intervention, during the second week of the washout phase, and at the end of second intervention. During each of the six week intervention periods, daily pain diaries were completed. Adverse events were recorded during each visit.

A timeline for all visits is presented in Additional file 1: Figure S1. At screening and final visits, a complete standardized neurological examination and full physical examination was carried out [13]. Each patient was scored using the Utah Early Neuropathy Scale (UENS) determining DPN severity [14]. After clinical scales were completed, candidates were verified to have DPN if the UENS score was ≥ 6. UENS scores of <6 led to exclusion due to uncertainty regarding DPN presence. Verification for PDPN presence was based upon the question “Do you have pain on a daily or near daily basis?” If affirmative, candidates then completed the Douleur Neuropathique (DN) 4 questionnaire (DN4Q), categorizing pain as NeP or non-NeP [15]. Candidates scoring ≥4 on the DN4Q were categorized as PDPN, otherwise exclusion occurred. Pain duration must have been ≥ 3 months with pain severity at least 40/100 mm on the visual analog scale (VAS) (Short-Form-McGill Pain Questionnaire) [16].

### Allocation and criteria

**Figure 1:**
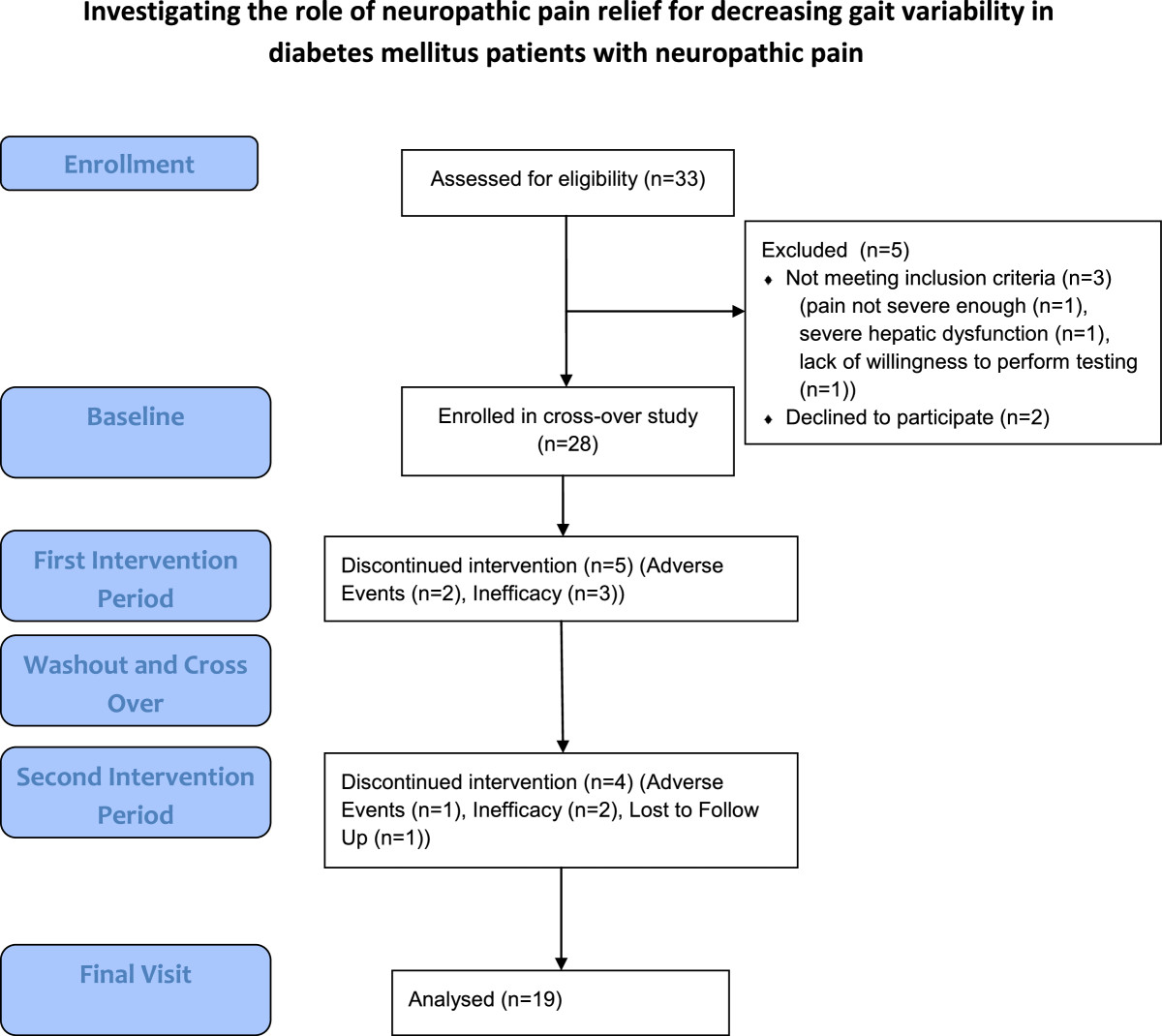
**A flowchart of subjects throughout the study using the CONSORT guidelines.**

A randomization table assigned subjects to the first cross-over intervention phase. Randomization concealment occurred for subjects, the clinical coordinator, and the study physician. Scheduled study portions were as follows: 1) Pre-screening to allow wash-out of any prohibited medications (see below); 2) a one week screening period to ensure subject eligibility into the study; 3) a six week randomized active treatment period (Intervention 1); 4) a two week washout period with weaning of pregabalin/placebo; 5) a six week randomized active treatment period (Intervention 2); and 6) a one week follow-up period with weaning of placebo/pregabalin (Figure 1). Telephone visits took place one week into each intervention period, and one week after the washout period to assess tolerability and adverse effects.

Inclusion and exclusion criteria are provided in Table 1. This was an adjuvant study - other pharmacotherapies for pain relief excluding gabapentinoids were permitted if stable from 1 month prior to study initiation to end of study. Prior use of pregabalin was permitted unless inefficacy or intolerability arose after appropriate dosing.

**Table 1:** **Inclusion and exclusion criteria for study entry are listed**

*Inclusion criteria*	1) Subjects aged ≥18 years
	2) Females of childbearing potential must have a negative urine pregnancy test performed, and all subjects were required to be practicing an effective form of contraception as required
	3) A diagnosis of NeP due to DPN with NeP severity of ≥4 on a Visual Analog Score
	4) Ability to complete pain diary and quality of life questionnaires and to perform GaitMeter™ and associated testing; and
	5) The subject must be willing and able to comply with scheduled study procedures.
*Exclusion criteria*	1) Another co-existing pain for which the subject or a qualified pain physician cannot differentiate from NeP due to DPN
	2) Clinically significant or unstable medical or psychological conditions
	3) Any history of malignancy, except either where there has been no ongoing treatment for at least 6 months or for a basal cell carcinoma
	4) A history of seizures, clinically significant cardiac arrhythmias, postural hypotension, uncontrolled hypertension, severe cardiovascular disease, severe hepatic impairment (evidence from medical history), renal failure or pulmonary disease
	5) A history of another diagnosed disorder which could interfere with gait and testing, such as with stroke, dementia, Parkinsonism, spinal cord disorder, muscle weakness, or use of a medication which causes significant sedation or incoordination
	6) Hepatic dysfunction at screening (aspartate transaminase [AST] or alanine transaminase [ALT] greater than twice the upper limit of normal, or a total bilirubin greater than the upper limit of normal
	7) Estimated creatinine clearance of < 60 mL/min based on the Cockcroft and Gault equation
	8) A positive urine drug screen
	9) A lack of willingness to discontinue and wash-out use of prohibited medications or treatments
	10) Inability to walk a total of 50 metres without any pauses or use of a cane, walker, or motorized device
	11) Identification of the presence of another potential cause for peripheral neuropathy, presence of impaired glucose tolerance only or juvenile onset of diabetes with requirement for insulin at time of diagnosis (i.e. possible type 1 diabetes)
	12) Refusal to perform concurrent laboratory and physiological testing.

### Pharmacological interventions

For pregabalin interventions, subjects were commenced on 75 mg po bid, with scheduled times to increase their dose once weekly after initiation to 150 mg po bid and then 300 mg po bid (maximum dose) if tolerated and agreed upon. A decrease in dose was permitted on one occasion to no less than 75 mg po bid. Tablets supplied were of 75 mg size. Placebo tablets were identical in appearance, size, color, taste, and smell and dosing.

### Outcome measures

GaitMeter™ analysis was performed prior to and after completion of 6 weeks for each pharmacological intervention. As described [9], GaitMeter™ is a small, wireless, portable, inertia-sensing motion analysis system using miniature accelerometers and gyroscopes attached to the anterior mid shin regions permitting various mobility tasks in a natural environment while data is continuously stored on a personal handheld computer. Subjects walked at normal pace for 50 metres using one 90 degree turn without rest time allowed. Data collection occurred between a few seconds before gait commencement until a few seconds after conclusion. Step length was measured individually for each leg and averaged in all cases using tibial acceleration, as analyzed using LabView^DM^ (National Instruments, Austin, TX) software. Although obtained, angular velocity measures were not studied due to the linear nature of the task studied with GaitMeter™. The first and last steps were not analyzed, as these are not typically a full step and would contribute to variability incorrectly if included. Step velocity was calculated using the distance and time of each individual leg’s step as measured by GaitMeter™. The degree of statistical variability in each of gait step distance and velocity was used to calculate measures of gait variability, defined as the extent to which the individual data diverged from the average value using variance. Although GaitMeter™ has been studied previously and found reliable, this device has not been validated against an established device or procedure for evaluation of gait in this manner. Secondary outcome measures included assessment of the mean difference in the average daily pain score, averaged from the final seven entries in the daily pain diary during the last week of the intervention period. Comparison was performed with average scores obtained in either the baseline week or in the last week of the washout period prior to each intervention period. Pain severity was rated daily using an 11-point numerical rating scale (NRS) from 0 = no pain to 10 = worst possible pain using an average score for the preceding 24 hours.

Secondary physiological outcome measures were performed at baseline week, at the last week of the washout period, and at the end of each intervention period. A Physiological Profile Approach (PPA) [17] was performed - this tests high and low contrast visual acuity, visual field dependence, peripheral sensation (tactile, vibratory, and proprioceptive sensation), muscle force using dynamometry, reaction time, and balance using standard clinical measures and complimentary measures. Corrected visual acuity testing was performed using a standardized Snellen eye chart. Proprioceptive testing was performed at the knee as described [17], with scoring in degrees of disparity between levels of the great toes. Contrast sensitivity was performed using the Vistech chart to determine orientation of gratings provided in rows of circles differing in contrast [18], scored as logarithmic values of inverses. Blood pressure measurements for lying, sitting, and standing were performed using one minute latencies. Mobile hand-held dynamometry using a Chatillon MSE100 dynamometer (Digital Measurement Metrology, Brampton, ON) for ankle dorsiflexion and plantar flexion [19] was performed. Reaction times of the dominant foot using a visual stimulus and recording of latency for the foot tapping on a measurement device. Balance testing used four scenarios to measure postural sway [17] – standing on floor with eyes open or closed, and standing on 15 cm thick foam with eyes open or closed, each for 30 seconds duration. Sway was calculated using a rod fixed to the posterior aspect of a belt while on the opposite end, a computer tablet pencil would scroll on touch-sensitive computer screen to measure percentages of movement based upon the computer screen area. Additional testing for determination of fall risk included the Timed Get-Up-and-Go Test (TUG) [20] and the Tinetti Assessment Tool (TAT) [21]. All subjects also completed the Modified Falls Efficacy Scale (MEFS) [22] and the Elderly Falls Screening Test (EFST) [23] at 0, 2, 6 and 8 weeks into each intervention.

All adverse events, both spontaneously reported and observed, were coded during each clinic or telephone visit using Medical Dictionary for Regulatory Activities (MedDRA) terminology. Vital signs and body weight were measured at clinic visits. Electrocardiograms were performed at screening and final visits.

### Analysis

The primary endpoint was defined as a measurable difference in the absolute gait variability using GaitMeter after 6 weeks of each intervention. Based upon the primary outcome measure, assuming a 2-sided alpha of 0.05 with a standard deviation of 0.02 (SD 0.02), a sample size was calculated for 25 patient subjects providing 90% power to avoid a type 1 error of <0.01 to ensure reliability. We anticipated a 10% drop out rate, and therefore 28 subjects were to be recruited.

The primary outcome measure analyzed was intra-subject variability in gait for step length and velocity considering all steps made by both limbs except for first and last steps of the 50 metre walk. Intra-subject variability calculations averages were obtained for all subjects during each intervention. Shapiro-Wilk testing was performed to ensure normal distributions prior to comparisons of variance for variables of interest. If normality was determined, then Bartlett-Box testing was performed to assess for homogeneity of variance between cohorts, using the null hypothesis that both interventions were associated with the same degree of variance for gait variables of interest. In the case of non-normal distributions for variance, general multivariate analysis of covariance (MANCOVA) testing using intra-subject variability in gait step length and step velocity as dependent measures with intervention was used as an independent measure. MANCOVA testing can be used when there are multiple dependent variables, yet significant differences between group means are sought. Each of the above tests were previously used in our work preliminary to this study [9]. Interlimb differences for each subject were compared for step length and velocity. Post-hoc linear regression was performed for determining relationships of gait variability with measures of step length, step velocity, and pain severity, using pain severity as a dependent variable, and step velocity and step length as independent or dependent variables depending upon the assessment performed. Post-hoc comparisons in the change in variability between baseline and endpoint timepoints have also been performed as a comparison between the two interventions administered. Absolute changes in variables were examined for significant differences for all variables examined at different time points. Separate ANOVA testing with calculation of F values was performed using Bonferroni corrections for the remaining secondary and tertiary outcome measures. α was set to be 0.05. Only data received from patients completing the entire study was analyzed. Analysis occurred using Microsoft Excel 2007 (Office 2007, Microsoft Corp, Washington State) or SPSS 14.0 (IBM, Armonk, NY). An intention-to-treat analysis could not be performed due to the primary outcome measure being dependent upon the gait assessments occurring at both the first and last week for each intervention.

## Results

### Participants

We recruited 28 subjects after assessing 33 potential candidates (Figure 1). A total of 19 subjects completed the entire study; discontinuations occurred in both intervention periods due to inefficacy of pain relief and intolerability, with one patient lost to follow-up for uncertain reasons (Table 2). There was no significant difference in the numbers of other pain medications used between baseline and endpoint times, or between the two interventions performed for either baseline and endpoint timepoints.

**Table 2:** **Subject characteristics**

	All study subjects (n = 28)	Subjects completing study (n = 19)	
Age	64.6 ± 10.4 years	65.7 ± 10.8 years	
Gender (males)	17/28 (61%)	16/19 (84%)	
Duration of Diabetes Mellitus	7.2 ± 3.9 years	7.5 ± 4.1 years	
Duration of Neuropathic Symptoms	5.7 ± 4.1 years	6.0 ± 4.3 years	
Concomitant Diabetic			
Complications Present (other than neuropathy)	5/28 (18%)	3/19 (16%)	
Other Neuropathic Pain Medications Used	1.2 ± 1.1	1.4 ± 1.2	
Weight (kg)	96.3 ± 26.8	94.6 ± 25.9	
Height (cm)	170.9 ± 7.6	169.7 ± 7.8	
Sitting Systolic Blood Pressure (mm Hg)	141 ± 18	139 ± 18	
Sitting Heart Rate (/min)	72 ± 10	74 ± 12	
Standing Systolic Blood Pressure (mm Hg)	140 ± 19	136 ± 20	
Standing Heart Rate (/min)	73 ± 12	73 ± 13	
Utah Early Neuropathy	16.3 ± 9.6	16.2 ± 6.5	
Score			
Douleur Neuropathique 4 Score	7.6 ± 1.0	6.9 ± 0.9	
		Block 1 Pregabalin Intervention	Block 1 Placebo Intervention
Baseline Visual Analog Pain Score		6.1 ± 1.6	7.1 ± 1.3
Average Pregabalin		205.8 ± 28.9	188.1 ± 39.8
Dosage Achieved (mg)			
Other Neuropathic Pain Medications Used		1.4 ± 1.2	1.4 ± 1.2

Values shown are means ± standard deviations.

Gaitmeter™ assessment revealed no significant differences in durations of time required to walk the entire 50 metres (Table 3) (ANOVA, p = NS, F = 0.3-0.7). Average actual step length and step velocity measures were unchanged between timepoints and interventions (ANOVAs, p = NS, F = 0.5-0.9). The baseline degrees of both step length and velocity variance varied between pregabalin and placebo intervention periods, with the placebo intervention demonstrating greater variability for both measures (Bartlett-Box tests, *χ*^2^ = 4.49-5.27, p = 0.022-0.034). In contrast to our primary hypothesis, the degree of variability in both step length and step velocity significantly increased for subjects receiving pregabalin for comparison of baseline and final visits (Bartlett-Box tests, *χ*^2^ = 5.13-9.27, p < 0.025). Although step length variability did not change during the placebo intervention (Bartlett-Box test, *χ*^2^ = 1.23,p = NS), step velocity variability decreased during the placebo intervention (Bartlett-Box test, *χ*^2^ = 17.07, p < 0.001). None of the cohorts had greater variability with one leg as compared to the other leg (Bartlett-Box tests, *χ*^2^ = 0.62-1.11, p = NS). Despite differences in variability, there were no significant differences between interventions with respect to step length or step velocity (MANOVAs, p = NS, F = 0.7-1.0). However, there was a difference in the change in variability from baseline to endpoint measurements for each of step length variability (Bartlett-Box test, *χ*^2^ = 6.22,p = 0.01) and step velocity variability (Bartlett-Box test, *χ*^2^ = 9.57, p = 0.001). Finally, there were no significant differences for individuals between the right and left legs for either of step length or step velocity variability (ANOVAs, p = NS, F = 0.2-0.3).

**Table 3:** **Results of gait testing**

Characteristic	Pregabalin intervention - baseline (n = 19)	Pregabalin intervention - final (n = 19)	Placebo intervention - baseline (n = 19)	Placebo intervention – final (n = 19)
Cadence (Steps/Minute)	137.0 ± 24.9	126.5 ± 24.2	137.4 ± 21.9	140.6 ± 22.9
Duration of Time to Walk 50 Metres (seconds)	39.8 ± 0.8	40.2 ± 1.1	39.7 ± 0.7	39.5 ± 0.9
Average Step Length (metres)	0.55 ± 0.11	0.59 ± 0.13	0.55 ± 0.15	0.54 ± 0.15
Average Step Duration (seconds)	0.44 ± 0.08	0.47 ± 0.09	0.44 ± 0.07	0.43 ± 0.07
Step Length Variance	0.060 ± 0.002	0.087 ± 0.010*	0.098 ± 0.005	0.100 ± 0.003
Step Velocity Variance	0.043 ± 0.001	0.066 ± 0.003*	0.058 ± 0.003^Ψ^	0.038 ± 0.000^κ^

Values shown are means ± standard deviations. MANOVA testing was used for step length/velocity variance comparisons. ANOVA testing was used for other measures shown. *p < 0.025 for comparison of baseline and final visit testing in the pregabalin intervention period. ^Ψ^p < 0.025 for comparison of baseline visit testing for the pregabalin and placebo intervention periods. ^κ^p < 0.025 for comparison of baseline and final visit testing in the placebo intervention period.

Subjects during the pregabalin intervention period received non-significant pain relief overall when compared to placebo (Additional file 2: Figure S2). There were no significant differences in pain scores at any timepoint based upon either change from baseline or for the cohort’s actual pain scores for each timepoint (multiple ANOVAs, p = NS, F = 0.6 – 3.7). Scoring for disruption of sleep due to pain had a similar non-significant representation (Additional file 3: Figure S3). Although the average score on the MEFS improved initially with pregabalin intervention, overall scoring representation was not significantly different between interventions (Additional file 4: Figure S4).

There were no significant differences for each of high and low contrast visual acuity, proprioceptive thresholds, and dynamometry-measured forces for leg muscles (ankle dorsiflexion and ankle plantar flexion) (Additional file 5: Table S1) or with balance testing, reaction times, TUG times, TAT scores, and EFST scores (Additional file 4: Figure S4). There were no changes in corrected visual acuity during the course of the study for any subject.

We used multiple linear regression analyses to identify positive associations between step length and step velocity (F = 12.5, R^2^ = 0.38, p < 0.0125), and for a negative relationship between step length and pain severity (F = 10.9, R^2^ = 0.33, p < 0.0125), but not for step velocity and pain severity (F = 7.1, R^2^ = 0.24,p = NS).

## Discussion

Not as hypothesized, subjects with PDPN receiving pregabalin treatment had increasing variance for both step length and step velocity. Surprisingly, subjects receiving placebo had the unanticipated result of a decrease in step velocity variance (but not for step length variance). These differences occurred without other noted differences, and we interpret these results as related to the interventions themselves. Also, it is important to note that the clinical importance of gait variability is unclear, and is not associated with any particular clinical and obvious outcome. Our results suggest that management of pain relief may not improve gait variability in PDPN subjects, although the pregabalin intervention in our trial did not provide significant pain relief limiting full interpretation of this trial’s results.

The presence of PDPN contributed to greater gait variability in our prior work [9], with potential contribution to postural instability and even falls. We hypothesized that pain itself contributed to this variability and development of an overly cautious antalgic gait with smaller steps. However, the addition of pregabalin may negatively impact gait function. Rats receiving extreme pregabalin doses have a dose-dependent impairment in rotarod performance [24]. Doses administered in our study were about 1000 fold less by weight, but the sensitivity of GaitMeter™ may be sufficient to identify small changes in human gait. Rats receiving phenytoin or carbamazepine are subject to much more profound ataxia [24]. Although ataxia was not reported as an adverse effect, it has been reported in addition to vertigo, nystagmus, incoordination and balance disorders as due to pregabalin [25], but with less magnitude than phenytoin and carbamazepine [26]. As such, it is possible that pregabalin’s adverse effects may have contributed to our findings.

There was a non-significant improvement with pain relief using pregabalin as a large confounder in this trial; if greater pain relief had occurred, it is possible that improved gait variability may have been observed. However, the discovery of placebo intervention’s association with reduced gait variability was unanticipated. In studies examining gait parameters in patients with knee osteoarthritis, placebo did not impact upon outcome measures [27]. The degree of placebo response is highly variable [28] and subject to particular personality types [29]. Other studies, however, have identified placebo-associated improvements in gait and balance [30]. Furthermore, subjects with low back pain are subject to reduced variability of gait [12], so perhaps the PDPN population has other distinct features leading to gait variability rather then the chronic pain itself.

Factors increasing gait dysfunction [31] include stride time variability, poor functional status or physiological capacity. Exercise programs may benefit instability [31]. Otherwise, management of gait dysfunction largely depends upon the disease process. Management of Parkinsonism and cardiovascular conditions, for example, improves gait dysfunction. Improvement in postural hypotension or discontinuation of medications causing ataxia or sedation can also improve gait. Fall prevention techniques are also of value, including gait assistance devices.

DPN patients have other contributing factors for falling [6] beyond NeP. Proprioceptive and cutaneous sensory loss also contributes. An antalgic gait in PDPN patients also contributes to instability [11]. Antalgic gait contributes to slow walking along with increased step frequency, reduced step length, less lateral sway in center-of-mass, and reduced amounts of ankle plantar flexion and hip extension movements, resulting in greater gait variability [9]. However, treatment of PDPN-associated NeP failed to reduce gait variability and, in fact, increased gait variability. This suggests that NeP management is unlikely to reduce risks of falling if of insignificant amount, but it remains unclear if significant pain relief may impact upon gait dysfunction.

Our findings should not dismay other research teams from identification of other potential risk factors for falling in DM. There are limitations to our results. The largest limitation was the absence of successful pain relief with pregabalin, either based upon change from baseline pain severity or as an absolute difference. This insufficient pain relief may have been due to a number of possible factors including a type II error, insufficient dosing, insufficient duration of treatment, or more refractory pain in our patient population. Also, the design of the study using both baseline and endpoint gait measures limited us from performance of an intention-to-treat analysis. The presence of greater baseline gait variability in the placebo intervention portions was unanticipated, and suggests that randomization may have been better performed using a stratified approach after baseline gait measures were performed. Calculation of foot contact moments and other potentially useful measures could not be performed using GaitMeter™. We did not formally assess cognitive function or vestibular function. Our tertiary care patients enrolled may not be representative of primary care populations. Although sample size calculations were met, discontinutations were more frequent than expected, dampening our results. Training effects over the four GaitMeter™ sessions performed may have influenced the results of the second intervention, and incomplete washouts may have occurred. GaitMeter™ has been shown reliable in this patient population based upon our prior work, but has not received validation with comparison against established gait analysis devices or procedures; as such, this invalidation may have contributed to the unanticipated results observed. Although other pain relief medications were stable, they may have impacted upon results. There were large degrees of variance in many examined measures, particularly with the baseline timepoint gait measures in the placebo intervention group. Finally, the lack of significant pain relief with pregabalin makes interpretation of these results difficult.

## Conclusion

Our results suggest that although PDPN impacts upon gait stability [9], its potential relief using pharmacotherapy may not improve gait dysfunction. Future studies with larger sample sizes and other forms of management for NeP should be considered. Although our hypothesis was not met, management of PDPN is clearly associated with other significant benefits related to pain relief, improved functionality and quality of life. We suggest that further kinesiological assessments of patients with PDPN are required to determine the reasons behind gait variability and its potential future management.

## Electronic supplementary material

Additional file 1: Figure S1: A timeline for all visits in this trial is presented. In person visits are demonstrated by V_1_, V_2_, etc. while telephone visits are not shown. During screening, informed consent is obtained. History and physical examination, along with electrocardiography, blood work, recording of number of falls, urine pregnancy testing and DN4 questionnaires are performed on visits marked with a plus sign (+). After the run in phase and at the time of randomization to either of pregabalin or placebo, gait and physiological assessments, along with performances of all other questionnaires are performed for visits shown with an asterisk (*). During the time of cross over to the other intervention, a 2 week washout period occurs during weeks 7–8. The final visit occurs two weeks after the completion of the second intervention (or with study dropout) as indicated. (TIFF 814 KB)

Additional file 2: Figure S2: The impact of interventions upon VAS pain severity levels. Over the time of the second intervention, the cohort receiving placebo followed by pregabalin had significantly better pain relief during the second intervention as compared to the cohort receiving pregabalin, then placebo (informal post-hoc analysis, ANOVA, p < 0.05) **(A)**. However, when subjects are grouped according to intervention received, there was a non-significant pain relief identified with the pregabalin intervention (ANOVA, p = NS) **(B)**. Values shown are means ± standard error. (TIFF 58 KB)

Additional file 3: Figure S3: The impact of interventions upon VAS sleep disturbance severity levels. As with the pain severity measure, the cohort receiving placebo followed by pregabalin had significant improvement in sleep disturbance during the second intervention as compared to the cohort receiving pregabalin followed by placebo (informal post-hoc analysis, ANOVA, p < 0.05) **(A)**. However, when grouped based upon intervention received, there was a non-significant impact upon sleep disturbance during the pregabalin intervention (ANOVA, p = NS) **(B)**. Values shown are means ± standard error. (TIFF 61 KB)

Additional file 4: Figure S4: Scores on the Modified Efficacy Falls Score were not significantly different between interventions (ANOVA, p = NS) **(A)**. Likewise, performance on balance testing did not change with either intervention and was similar for all time points measured (multiple ANOVAs, p = NS) **(B)**. Reaction times were unchanged for any of the studied timepoints also (ANOVA, p = NS) **(C)**. Other assessments of mobility, including the Tinetti Up and Go time **(D)**, Tinnetti Assessment Tool **(E)**, and the Elderly Fall Screening Test score **(F)** were also unchanged between interventions (multiple ANOVAs, p = NS). (TIFF 1 MB)

Additional file 5: Table S1: Results of ancillary testing. (DOCX 18 KB)
