# Supplementary figures and images for: Investigating the role of neuropathic pain relief in decreasing gait variability in diabetes mellitus patients with neuropathic pain: a randomized, double-blind crossover trial
Source: J Neuroeng Rehabil. 2014 Aug 20;11:125. doi: 10.1186/1743-0003-11-125 (PMC4150964; doi:10.1186/1743-0003-11-125)

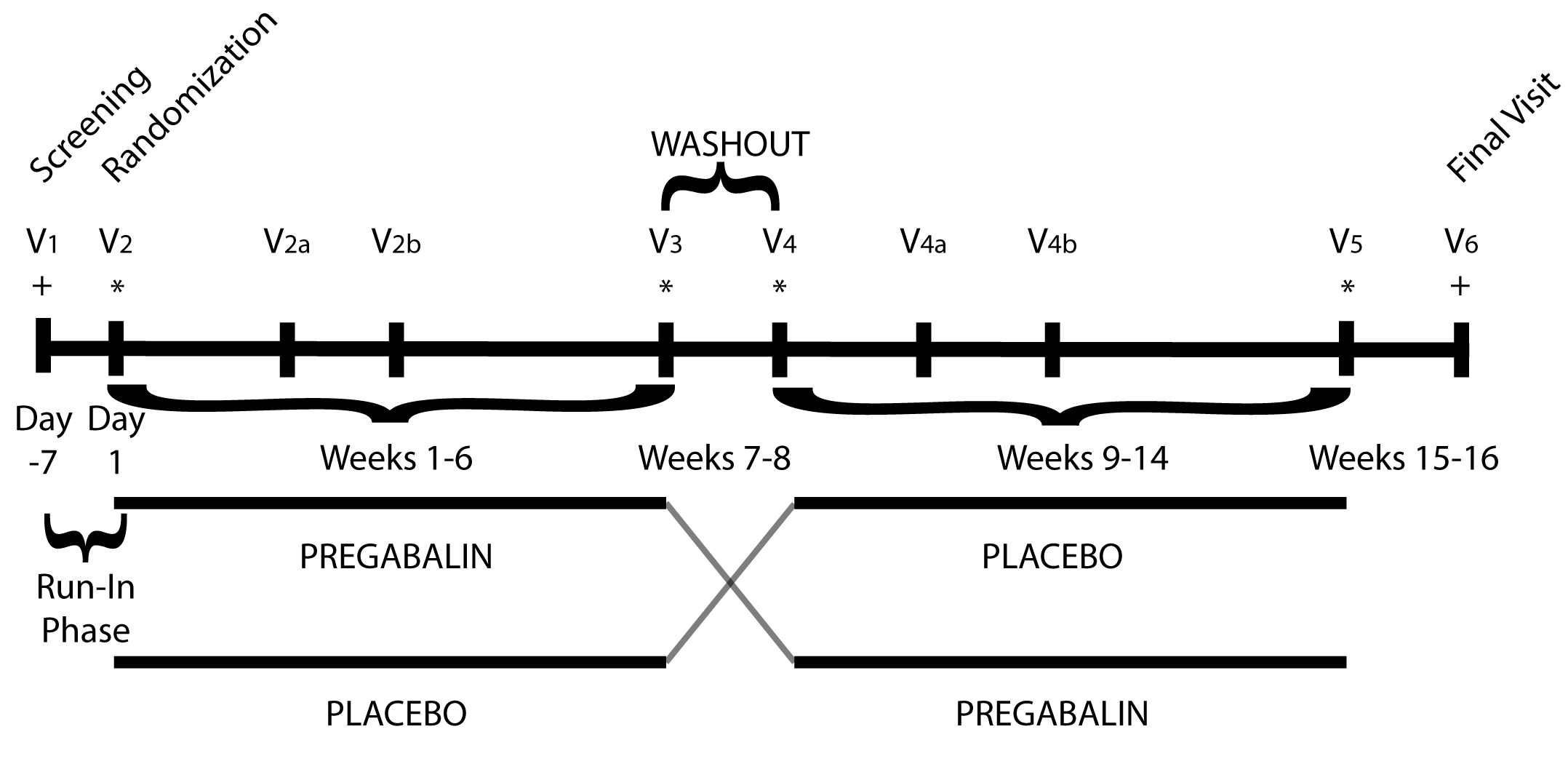

Supplement: Supplementary file 1 — Additional file 1: Figure S1: A timeline for all visits in this trial is presented. In person visits are demonstrated by V1, V2, etc. while telephone visits are not shown. During screening, informed consent is obtained. History and physical examination, along with electrocardiography, blood work, recording of number of falls, urine pregnancy testing and DN4 questionnaires are performed on visits marked with a plus sign (+). After the run in phase and at the time of randomization to either of pregabalin or placebo, gait and physiological assessments, along with performances of all other questionnaires are performed for visits shown with an asterisk (*). During the time of cross over to the other intervention, a 2 week washout period occurs during weeks 7–8. The final visit occurs two weeks after the completion of the second intervention (or with study dropout) as indicated. (TIFF 814 KB) [file 12984_2014_647_MOESM1_ESM.tiff]

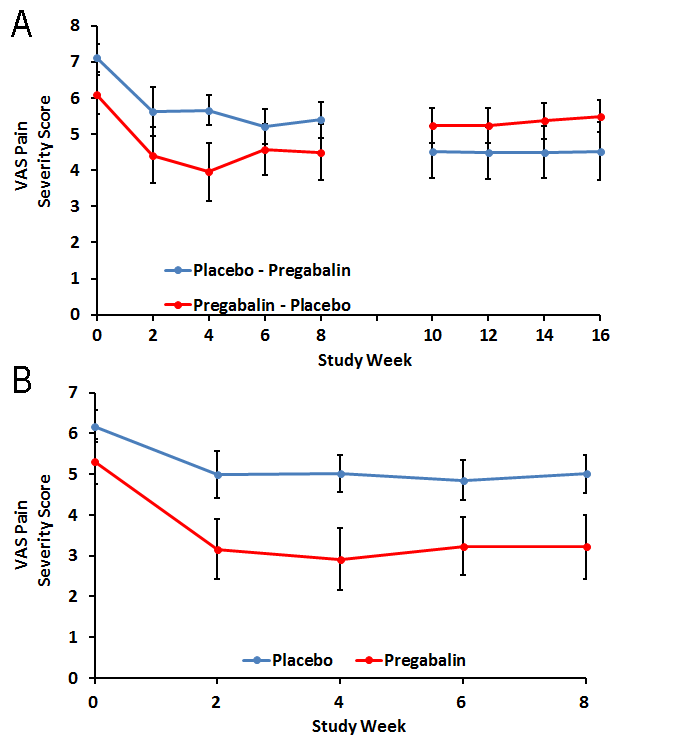

Supplement: Supplementary file 2 — Additional file 2: Figure S2: The impact of interventions upon VAS pain severity levels. Over the time of the second intervention, the cohort receiving placebo followed by pregabalin had significantly better pain relief during the second intervention as compared to the cohort receiving pregabalin, then placebo (informal post-hoc analysis, ANOVA, p < 0.05) (A). However, when subjects are grouped according to intervention received, there was a non-significant pain relief identified with the pregabalin intervention (ANOVA, p = NS) (B). Values shown are means ± standard error. (TIFF 58 KB) [file 12984_2014_647_MOESM2_ESM.tiff]

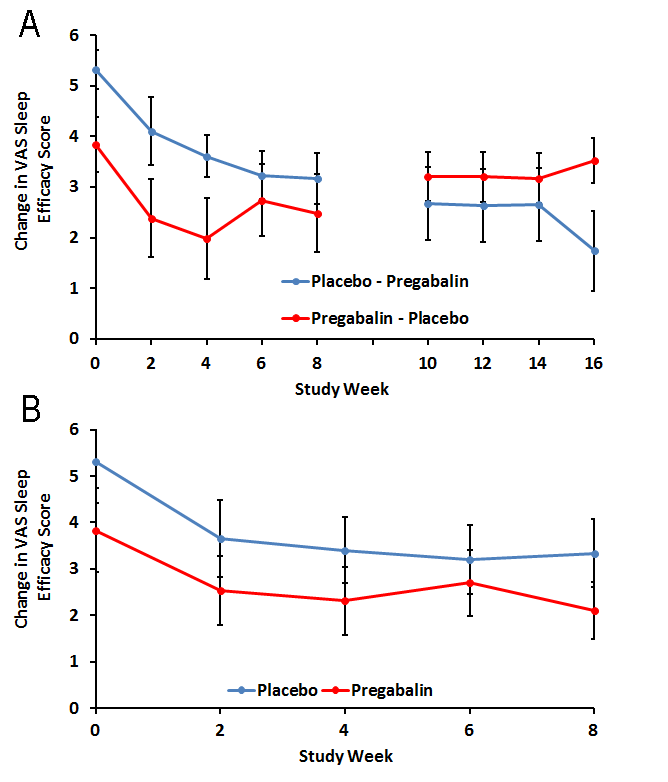

Supplement: Supplementary file 3 — Additional file 3: Figure S3: The impact of interventions upon VAS sleep disturbance severity levels. As with the pain severity measure, the cohort receiving placebo followed by pregabalin had significant improvement in sleep disturbance during the second intervention as compared to the cohort receiving pregabalin followed by placebo (informal post-hoc analysis, ANOVA, p < 0.05) (A). However, when grouped based upon intervention received, there was a non-significant impact upon sleep disturbance during the pregabalin intervention (ANOVA, p = NS) (B). Values shown are means ± standard error. (TIFF 61 KB) [file 12984_2014_647_MOESM3_ESM.tiff]

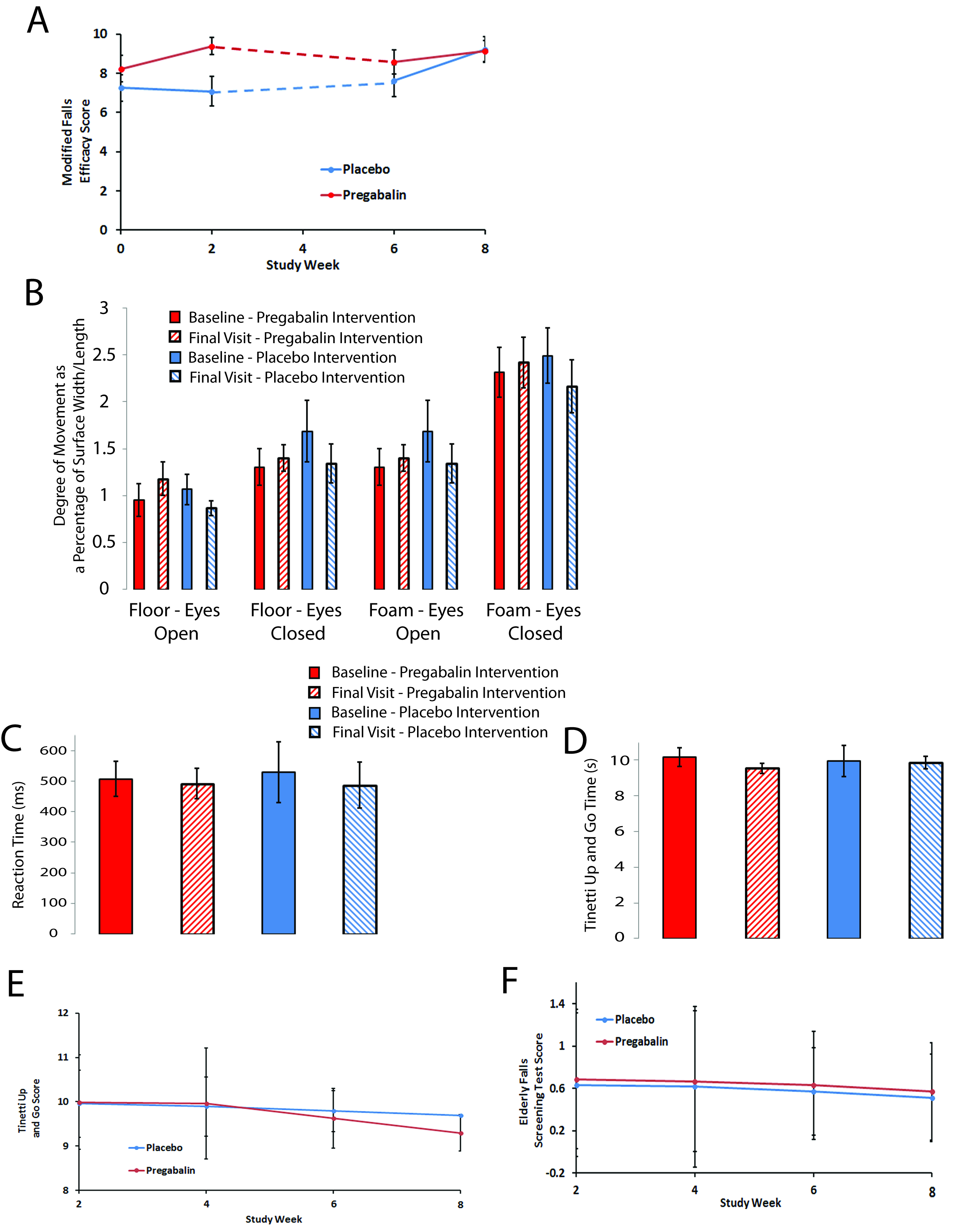

Supplement: Supplementary file 4 — Additional file 4: Figure S4: Scores on the Modified Efficacy Falls Score were not significantly different between interventions (ANOVA, p = NS) (A). Likewise, performance on balance testing did not change with either intervention and was similar for all time points measured (multiple ANOVAs, p = NS) (B). Reaction times were unchanged for any of the studied timepoints also (ANOVA, p = NS) (C). Other assessments of mobility, including the Tinetti Up and Go time (D), Tinnetti Assessment Tool (E), and the Elderly Fall Screening Test score (F) were also unchanged between interventions (multiple ANOVAs, p = NS). (TIFF 1 MB) [file 12984_2014_647_MOESM4_ESM.tiff]
