# Supplementary material for: Investigating the role of neuropathic pain relief in decreasing gait variability in diabetes mellitus patients with neuropathic pain: a randomized, double-blind crossover trial
Source: J Neuroeng Rehabil. 2014 Aug 20;11:125. doi: 10.1186/1743-0003-11-125 (PMC4150964; doi:10.1186/1743-0003-11-125)
Supplement: Supplementary file 5 — Additional file 5: Table S1: Results of ancillary testing. (DOCX 18 KB) [file 12984_2014_647_MOESM5_ESM.docx]

Additional file 5: Table S1 – Results of Ancillary Testing

|  |  |  | |  | |  | |  | |  |
| --- | --- | --- | --- | --- | --- | --- | --- | --- | --- | --- |
| Characteristic | Pregabalin Intervention - Baseline (n=19) | | Pregabalin Intervention - Final (n=19) | | Placebo Intervention - Baseline (n=19) | | Placebo Intervention – Final (n=19) | |  |  |
| Proprioception Disparities (Degrees) | 0.9±1.1 | | 0.7±0.8 | | 0.8±1.0 | | 0.8±1.1 | |  |  |
| Dynamometry Power (N·m)  Ankle Dorsiflexion  Ankle Plantar Flexion | 13.4±2.3  39.5±9.6 | | 12.7±1.7  38.2±10.7 | | 12.5±2.9  39.7±8.6 | | 13.5±1.4  40.2±7.7 | |  |  |
| Contrast Sensitivity (Spatial Frequency in Cycles Per Degree as Inverse Logs)  1.5  3  6  12  18 | -2.12±1.13  -2.23±1.26  -1.78±1.15  -1.13±0.93  -0.85±0.83 | | -2.11±1.16  -2.21±1.24  -1.67±1.01  -0.95±0.64  -0.79±0.64 | | -2.08±1.00  -2.18±1.19  -1.72±0.91  -0.99±0.73  -0.72±0.58 | | -2.03±1.02  -2.20±1.12  -1.67±0.99  -0.86±0.49  -0.67±0.31 | |  |  |

Values shown are means ± standard deviations.
